# *In-vivo* vascular application via ultra-fast bioprinting for future 5D personalised nanomedicine

**DOI:** 10.1038/s41598-020-60196-y

**Published:** 2020-02-21

**Authors:** Ruben Foresti, Stefano Rossi, Silvana Pinelli, Rossella Alinovi, Corrado Sciancalepore, Nicola Delmonte, Stefano Selleri, Cristina Caffarra, Edoardo Raposio, Guido Macaluso, Claudio Macaluso, Antonio Freyrie, Michele Miragoli, Paolo Perini

**Affiliations:** 1 Department of Medicine and Surgery, University of Parma, via Gramsci 14, 43126 Parma, IT Italy; 2 CERT, Centre of Excellence for Toxicology Research, via Gramsci 14, 43126 Parma, IT Italy; 3 Department of Engineering and Architecture, University of Parma, Parco Area delle Scienze, 43124 Parma, IT Italy; 4 Unit of Surgical Sciences, Azienda Ospedaliero-Universitaria, via Gramsci 14, 43126 Parma, IT Italy; 5 Centro Universitario di Odontoiatria, University of Parma, Via Gramsci 14, 43126 Parma, IT Italy; 6 IMEM-CNR National Research Council, Parco Area delle Scienze 37/A, 43124 Parma, IT Italy; 7 Unit of Vascular Surgery, Azienda Ospedaliero-Universitaria, via Gramsci 14, 43126 Parma, IT Italy; 8 Humanitas Clinical and Research Centre, via Manzoni 56, 20090 Rozzano Milan, IT Italy

**Keywords:** Biotechnology, Health care

## Abstract

The design of 3D complex structures enables new correlation studies between the engineering parameters and the biological activity. Moreover, additive manufacturing technology could revolutionise the personalised medical pre-operative management due to its possibility to interplay with computer tomography. Here we present a method based on rapid freeze prototyping (RFP) 3D printer, reconstruction cutting, nano dry formulation, fast freeze gelation, disinfection and partial processes for the 5D digital models functionalisation. We elaborated the high-resolution computer tomography scan derived from a complex human peripheral artery and we reconstructed the 3D model of the vessel in order to obtain and verify the additive manufacturing processes. Then, based on the drug-eluting balloon selected for the percutaneous intervention, we reconstructed the biocompatible eluting-freeform coating containing 40 nm fluorescent nanoparticles (NPs) by means of RFP printer and we tested the *in-vivo* feasibility. We introduced the NPs-loaded 5D device in a rat’s vena cava. The coating dissolved in a few minutes releasing NPs which were rapidly absorbed in vascular smooth muscle cell (VSMC) and human umbilical vein endothelial cell (HUVEC) *in-vitro*. We developed 5D high-resolution self-dissolving devices incorporating NPs with the perspective to apply this method to the personalised medicine.

## Introduction

Chronic lower extremity peripheral arterial disease (PAD) is a manifestation of systemic atherosclerosis, and one of the main causes in loss of walking ability. Two treatments for lower extremity PAD are currently available: surgical (primarily bypass surgery) and endovascular treatment (EVT) mainly through balloon angioplasty. In fact, due to its clear advantages at least in the short-term, EVT represents – at present – the most common treatment for PAD^1^. Drug-Coated Balloons are made up of a semi-compliant or non-compliant balloon catheter covered with an anti-proliferative agent (typically paclitaxel), and an excipient (e.g. urea) to facilitate drug transfer into the vessel wall after balloon inflation^2^. The BASIL (Bypass Versus Angioplasty for Severe Ischemia of the Leg) trial is the only multicentre, randomised and controlled trial (RCT) which compared these two treatments^3^. One of the major limitations of this RCT is that current endovascular technologies have not been included^4^. Important advances developed in endovascular technologies address a great variety of anatomic challenges, while current and future efforts are directed towards improving long-term patency rates.

The 3D printing techniques demonstrate the high potentiality of interactive processes applied to medicine and associated toxicity/vitality studies^5,6^. Materials selected for bio-printed scaffolds are predominantly based on both naturally derived polymers (including gelatine, collagen, alginate, chitosan and hyaluronic acid) or synthetic molecules (polyethylene glycol)^7–9^, allowing precise control^10^ of chemical and physical properties^11^.

The 5D printing merges data used to create 3D models with data regarding physiological activity for personalised therapy^12^. In the present work, we describe how the 5D additive manufacturing method is applied to create personalised models of patients’ pathology. In detail, we used available 3D printing technology to identify the operative processes^13^ and the related parameters able to reach the best fit between the digital model and final object. Thus, we selected bio-inks and new composite materials to satisfy the final functional application (4D). Finally, we customised macro/micro morphologies and biological properties (5D), merging the engineered devices with the shape obtained from the patient. The subsequently drug integration and *in vivo* analysis enable the implementation of the personalised medicine.

## Results

The 5D printing technique^12^ incorporates data regarding 3D printing technologies with local control composition of the biomimetic materials (+1D), and particles distributions^14^ able to reproduce the real organ response during the physiology studies (+1D). We reconstructed the 3D model from patient’s CT obtaining the nano-laden aerogel for the fast release of nanoparticles (NPs). The bioprinted devices were used to realise a biomimetic bio-composite material (4D) for the *in vitro* and *in vivo* tests. Finally, customising the 4D model using particles that directly interact with the organ physiology we obtained a 5D bioprinted device.

The design of a standardised approach, to develop 5D printed devices, required 3 phases (Table 1): pre-printing, printing and post-printing^15^. Each phase requires the analysis and the validation of the following modelling steps: i) requirement, ii) model orientation, iii) trajectory generation, iv) printing process analysis and v) digital model adherence^16^.

**Table 1:** Organ printing schema and FANNAM method (from pre-processing reconstruction to 5D customisation) describing the used methods, the parameters, the operative processes for each organ printing phases.

METHOD	PARAMETERS	OPERATIVE PROCESSES	PRINTING PHASES	ORGAN PRINTING PHASES
PARAMETERS IDENTIFICATION	Region of interest	Imaging acquisition (CT, MRI, PET)	Images processing sectioning and cell isolation	PRE-PRINTING
MATERIALS SELECTION	Process	Technological sizing based on the material properties	Formulation	
TECHNOLOGY ASSESSMENT	Geometrical	CATE (computer aided tissue engineering) processing	Blueprint	
PRE-PROCESSING RECONSTRUCTION	Printing	3D imaging digitization and printing simulation	3DRC (3D reconstruction cutting) and digital bio-library related to the model	
3D OBJECT PROCESSING	Chemical and physical	Additive manufacturing and fixation	3DFFG (3D fast freeze gelation)	PRINTING
4D FUNCTIONALISATION	Functional and biomimicry	Definition of dynamic and active properties	NDF (nano dry formulation)	
5D CUSTOMISATION	Pathology and physiology	Health device development	3DPP (3D partial processes) and digital bio-library functional customisation	
THERAPY	Predictive	Biological tests	Biomonitoring	POST-PRINTING
SYNTHETIC ORGAN TRANSPLANTATION	Health guideline	Clinical analysis	Treatment and validation	
BIO-BASED SMART BIOPRINTING	Adaptive	Smart processing	Human mimicry and autonomous regeneration	

Using a Peltier-based system (Supplementary Fig. S1a), we developed soft scaffolds (Supplementary Fig. S1b) with complex shape (Supplementary Fig. S1c). The implemented cooled bed (Supplementary Fig. S1d) demonstrated the ability to reach −30 °C in 19.6 ± 0.9 s (31.2 ± 0.1 °C in 120 s) and return to room temperature (ΔT = 57 °C) in 25.4 ± 0.7 s.

**Figure 1:**
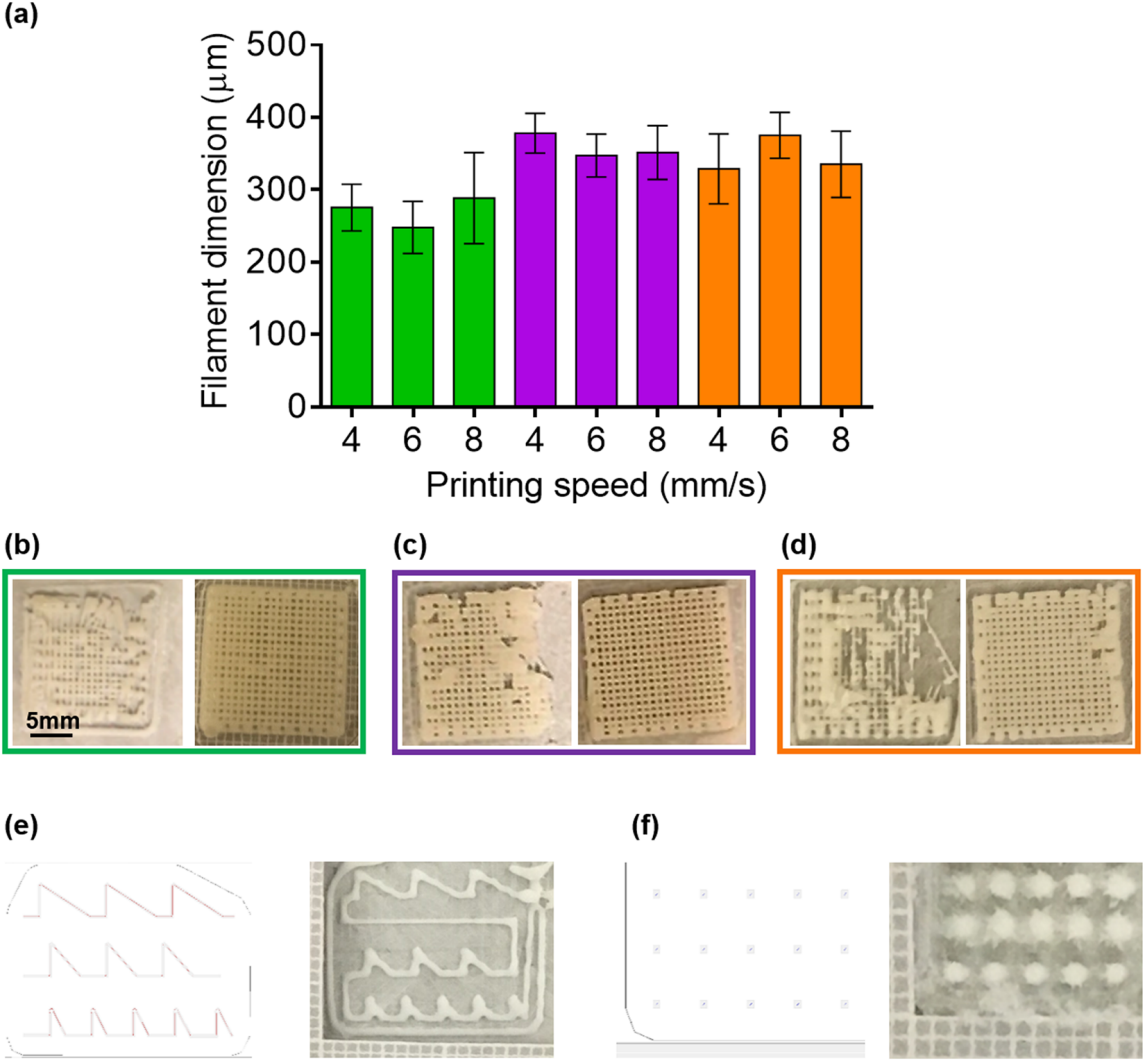
Scaffold macro-morphological characterisation and resolution assessment. (**a**) Scaffold filament diameter at different speed and alginate concentration with volume integration: 11% (green), 9% (purple), 7% (orange); (**b**) Printed scaffolds with 11% of alginate concentration under (left panel) and over (right panel) the minimum speed (6 mm/s). (**c**) same as (**b**) for printed scaffolds with 9% of alginate concentration and minimum speed of 10 mm/s. (**d**) same as (**b**) for printed scaffolds with 7% of alginate concentration and minimum speed of 14 mm/s. (**e**) Example of validation tests of printed corners at 60°, 45° and 30°:.STL file (left panel); printed trajectory (right panel). (**f**) Example of validation tests of printed drops:.STL file (left panel); printed trajectory (right panel). Wilcoxon sign rank test was performed and statistical significance was set at p < 0.05. Data are represented as median ± discrepancy.

The selected mechanical micro-extrusion technology^17,18^, with volumetric planning, did not require any process change in the use of different materials or bio-ink viscosities (Supplementary Fig. S2a). This is known to be impossible by pneumatic control extrusion without real-time pressure adaptation (Supplementary Fig. S2b–d). The fresh filament diameter, measured after 3D printing, demonstrated that 11% of alginate (Fig. 1a, green columns) was the optimal biomaterial concentration to obtain dimensions very close to the designed value (Φ = 292 µm, inner diameter of 26G syringe needle). In fact, using 7% and 9% alginate solutions in the fabrication of a scaffold with 400 µm of macro-porosity it is very challenging to maintain the bridge for the required freezing time. Thus, it is required a low environmental temperature to subtract the thermal energy in less time or in the case instead, to maintain the same speed, we have to enlarge the filament diameter with the final aim to better distribute filament weight. The minimum deposition speeds for the different percentages of biomaterial, assuring regular shape distribution, were 6 mm/s for 11% of alginate (Fig. 1b), 10 mm/s for 9% of alginate (Fig. 1c) and 14 mm/s for 7% of alginate. Finally, with the minimum speed (6 mm/s) and maximum concentration (11% of alginate) that we used for 3D bioprinting, we also performed a validation test of both corners and drop (Fig. 1e,f), demonstrating the high resolution of the applied technology.

**Figure 2:**
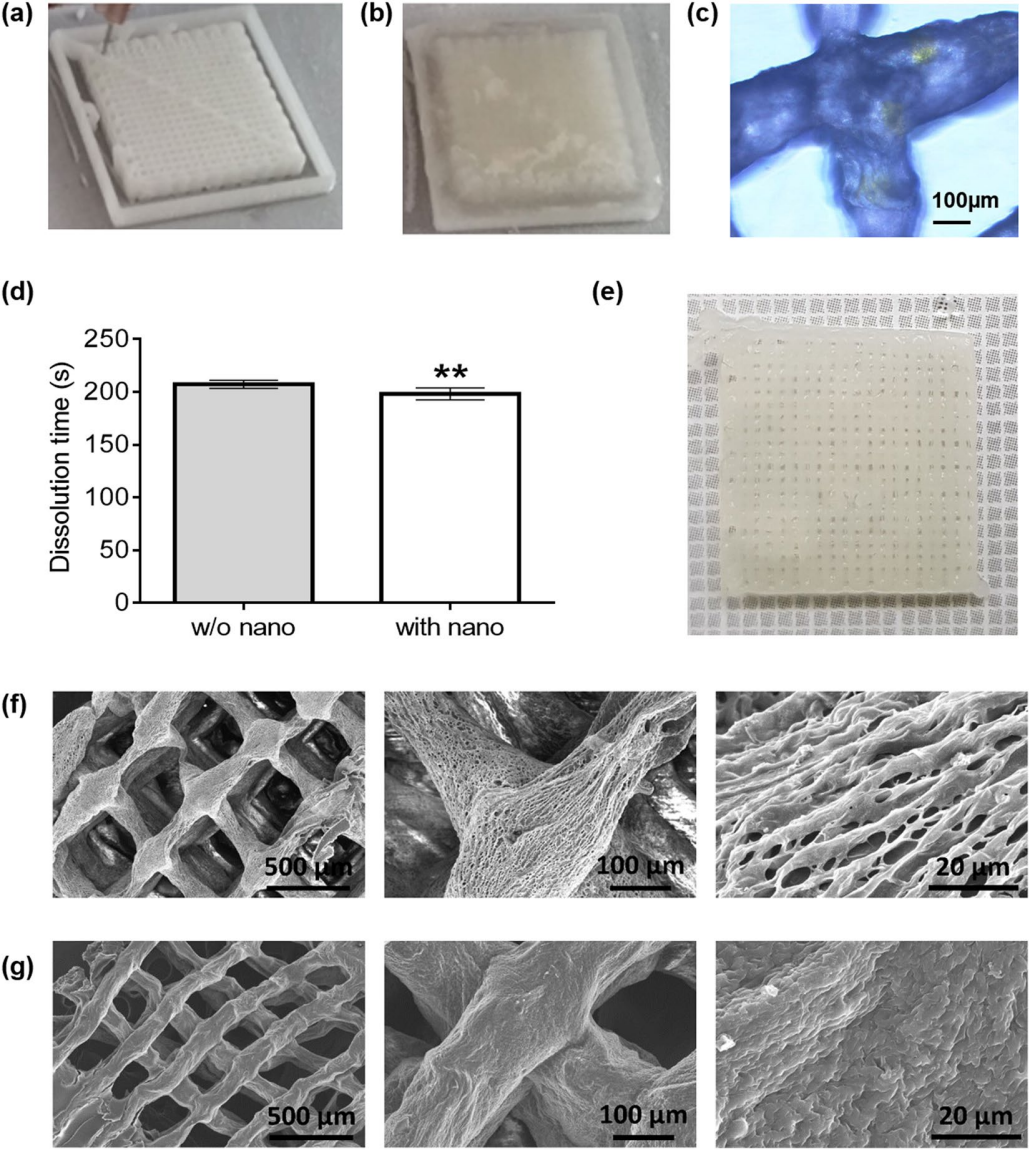
3D Fast Freeze Gelation, dissolution time and micro-morphology. (**a**) Freeze fixation; (**b**) Freeze gelation; (**c**) Scaffold detail displaying fluorescence spots. (**d**) Dissolution time of alginate ethanol gelled scaffold in DMEM, without (grey) or with (white) nanoparticles. (**e**) Alginate based scaffold (gelled with CaCl_2_) after 24 h conservation in DMEM. (**f**) Alginate SEM images of 3D printing scaffold gelled with CaCl_2_ and with (**g**) ethanol at different magnifications. Unpaired t-test was performed and statistical significance was set at p < 0.05. ** vs without nanoparticles. Data are represented as mean ± SEM.

We developed the fast release therapy through the bioprinting fixation (Fig. 2a) followed by freeze gelation, using ethanol^19^ (Fig. 2b), enabling the fabrication of 5D nano-laden hydrogel (Fig. 2c). We printed eight scaffolds with and without 40 nm fluorescent NPs (Fig. 2d), the scaffold dissolved in Dulbecco’s Modified Eagle’s Medium (DMEM), in 198.3 ± 1.6 s and 207.3 ± 2 s respectively, unlike the typical CaCl_2_ gelation scaffold (Fig. 2e) that displays the same structure after 24 h. Accordingly to scanning electron microscopy (SEM) images, the scaffolds printed with alginate showed an extremely porous and fibrillary microstructure. The high surface area led to a greater interaction with biological tissues (Fig. 2f). On the contrary, structures gelled with ethanol (Fig. 2g) showed a more compact and less porous microstructure, characterised by a higher speed of resorption, release of drugs and reduced surface volume ratio^20^.

**Figure 3:**
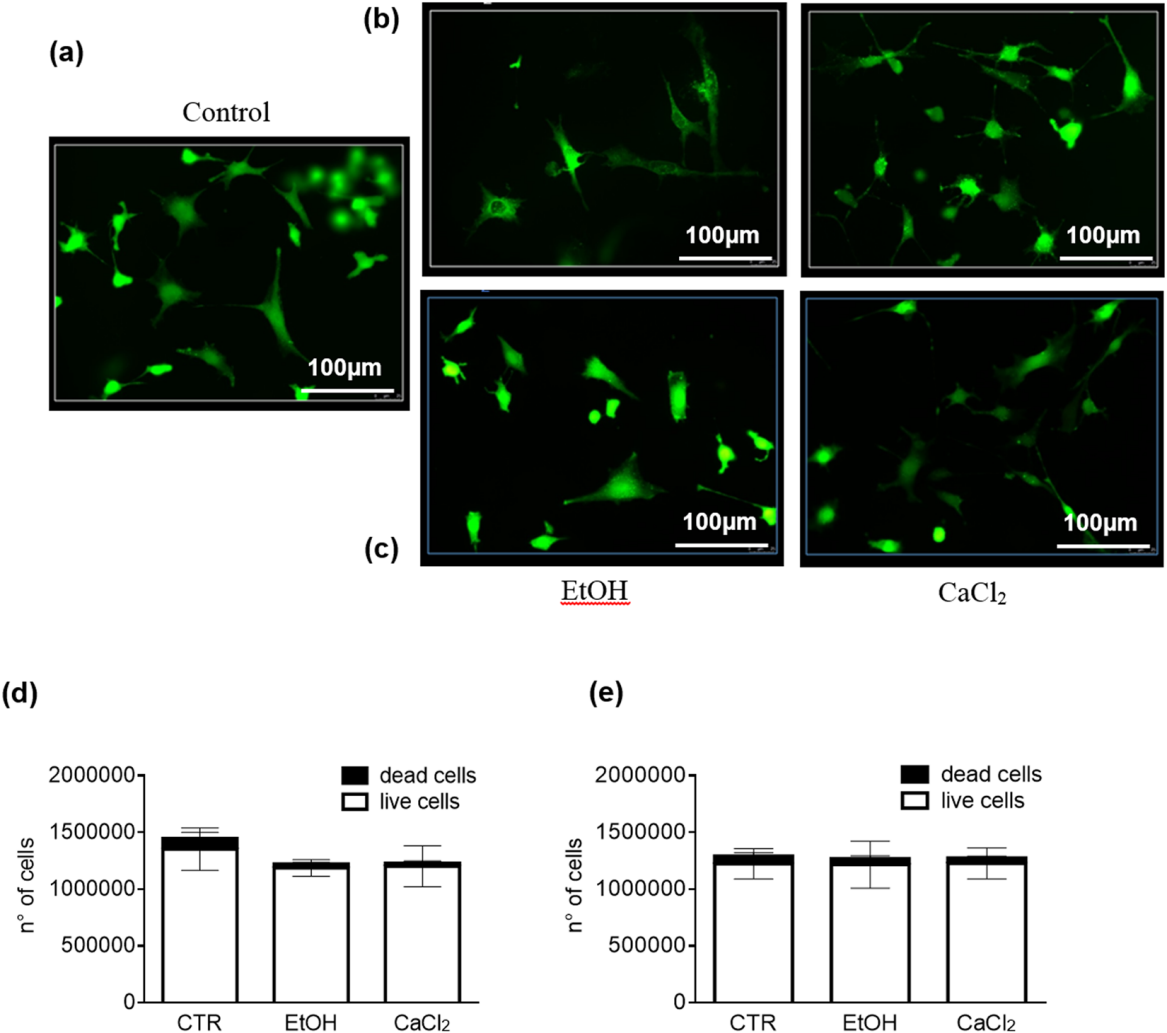
VSMC Viability. (**a**) Calcein AM-loaded VSMC after scaffold solubilisation with (**b**) direct or (**c**) indirect method, gelled with EtOH (left panels) or CaCl_2_ (right panels). VSMC viability (Live/Dead assay) after dissolving the scaffold, gelled with EtOH or CaCl_2_, with (**d**) direct or (**e**) indirect method. Unpaired t-test was performed and statistical significance was set at p < 0.05. Data are represented as mean ± SEM.

Preliminary *in-vitro* analysis on vascular smooth muscle cells (VSMCs) (Fig. 3), demonstrated that cell viability in both the gelation processes and dissolution (direct and indirect methods) did not change, when compared to control.

**Figure 4:**
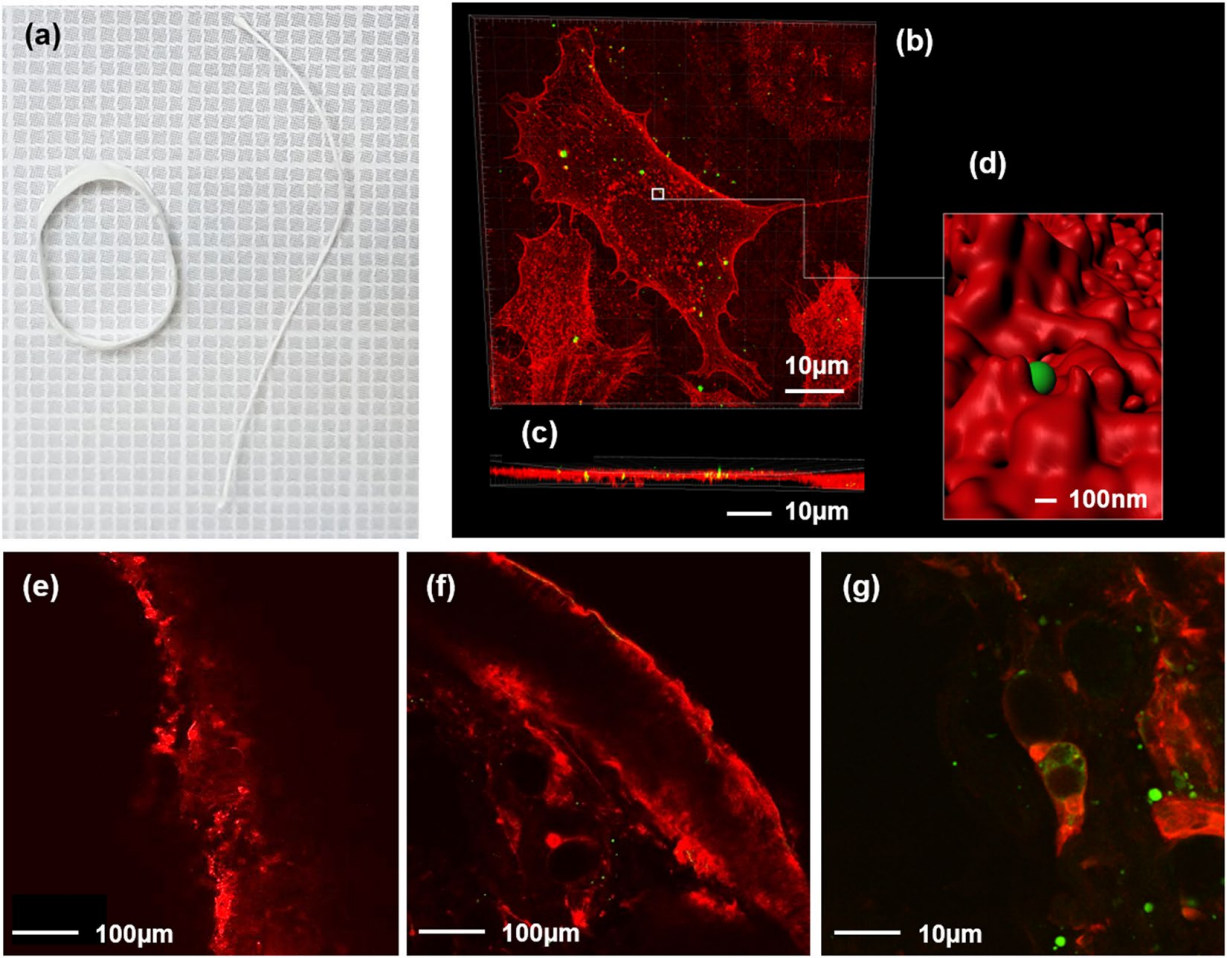
Scaffold-derived nanoparticles internalization. (**a**) Aerogel scaffold and filament after more than 12 months of storage in the petri dish. (**b**) Frontal view obtained by STED confocal microscopy of wheat germ agglutinin (WGA) stained vascular smooth muscle cells (VSMC, red) that included nanoparticles (green). (**c**) Orthogonal view showing the same nanoparticles into the cultured cells. (**d**) Render image obtained by the white square in “*b”* showing nanoparticle internalization from the VMSC membrane. Nanoparticle diameter: 40 nm. (**e**) Two-photon microscopy imaging of a WGA-stained rat vein. (**f**) Same as (**e**) with a vein exposed to the scaffold containing nanoparticles, showing the internalization of the nanoparticles in the VSMC cells. (**g**) same as (**f**) with high-scan resolution.

Furthermore, we observed NPs internalisation into cells, following dissolution of the structures (Fig. 4a) in VSMC thickness via stimulation-emission depletion (STED) (Fig. 4b–d) confocal microscopy. To confirm the feasibility^21^ of these methods *in-vivo*, we inserted the same NPs-laden structures in rat’s vena cava and we observed, after fast dissolution, NPs internalisation into both the interstitial tissue and the vascular cells (Fig. 4e–g).

**Figure 5:**
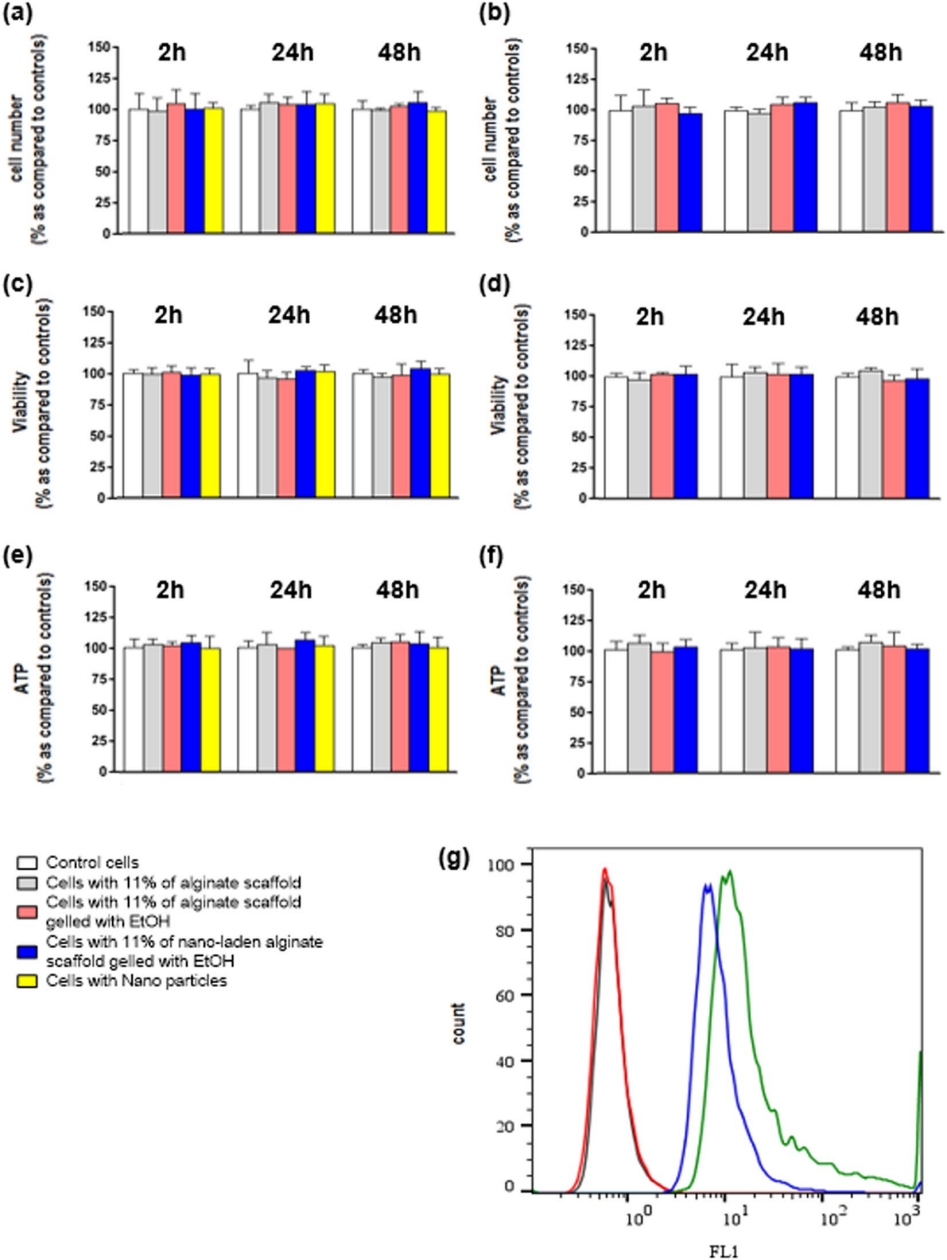
*In-vitro* viability and metabolism evaluation in HUVEC line. (**a**) cell number evaluation at 2 h, 24 h and 48 h after scaffold direct dissolution in: (i) control cell (first column); (ii) cells after 11% of alginate administration (second column); (iii) cell after 11% of alginate administration and ethanol crosslinking (third column); (iv) cell after 11% of alginate administration, ethanol crosslinking and NPs administration (forth column); (v) cell after NPs administration (fifth column). (**b**) same as (**a**) for scaffold indirect dissolution. (**c**) cell viability evaluation at 2 h, 24 h and 48 h after scaffold direct dissolution in: i) control cell (first column); (ii) cells after 11% of alginate administration (second column); iii) cell after 11% of alginate administration and ethanol crosslinking (third column); (iv) cell after 11% of alginate administration, ethanol crosslinking and NPs administration (forth column); (v) cell after NPs administration (fifth column). (**d**) same as (**c**) for scaffold indirect dissolution. (**e**) ATP evaluation at 2 h, 24 h and 48 h after scaffold direct dissolution in: (i) control cell (first column); (ii) cells after 11% of alginate administration (second column); iii) cell after 11% of alginate administration and ethanol crosslinking (third column); (iv) cell after 11% of alginate administration, ethanol crosslinking and NPs administration (forth column); (**v**) cell after NPs administration (fifth column). (**f**) same as (**e**) for scaffold indirect dissolution. White columns: control cells; grey columns: cells with 11% of alginate scaffold; pink columns: cells with 11% of alginate scaffold gelled with EtOH; blue columns: cells with 11% of alginate scaffold gelled with EtOH containing NPs: yellow columns: cells with NPs. (**g**) Cytofluorimetric analysis of HUVEC control cell after 24 h (black trace), HUVECs and alginate after 24 h (red trace), HUVECs and alginate plus NPs after 2 h (green trace) and after 24 h (blue trace). Kruskal-Wallis (post hoc analyses: Dunn’s multiple comparison) was performed and statistical significance was set at p < 0.05. Data are represented as mean ± SEM.

Finally, cellular viability and metabolism were evaluated in human umbilical vein endothelial cell (HUVEC) line in order to understand if each step of our printing method can alter cell surviving. It was evident that the percentage of alginate (11%), that we used for our biological application, did not influence cellular viability and metabolism in both direct (Fig. 5a,c,e) and indirect (Fig. 5b,d,f) dissolution. Moreover, gelation and NPs administration did not alter the HUVECs viability and metabolism (Fig. 5a–f). To confirm if NPs interact with our cell line, we also performed cytofluorimetric analysis showing that HUVEC are capable to bind and/or internalise NPs with little changes after 24 h of administration (Fig. 5g).

**Figure 6:**
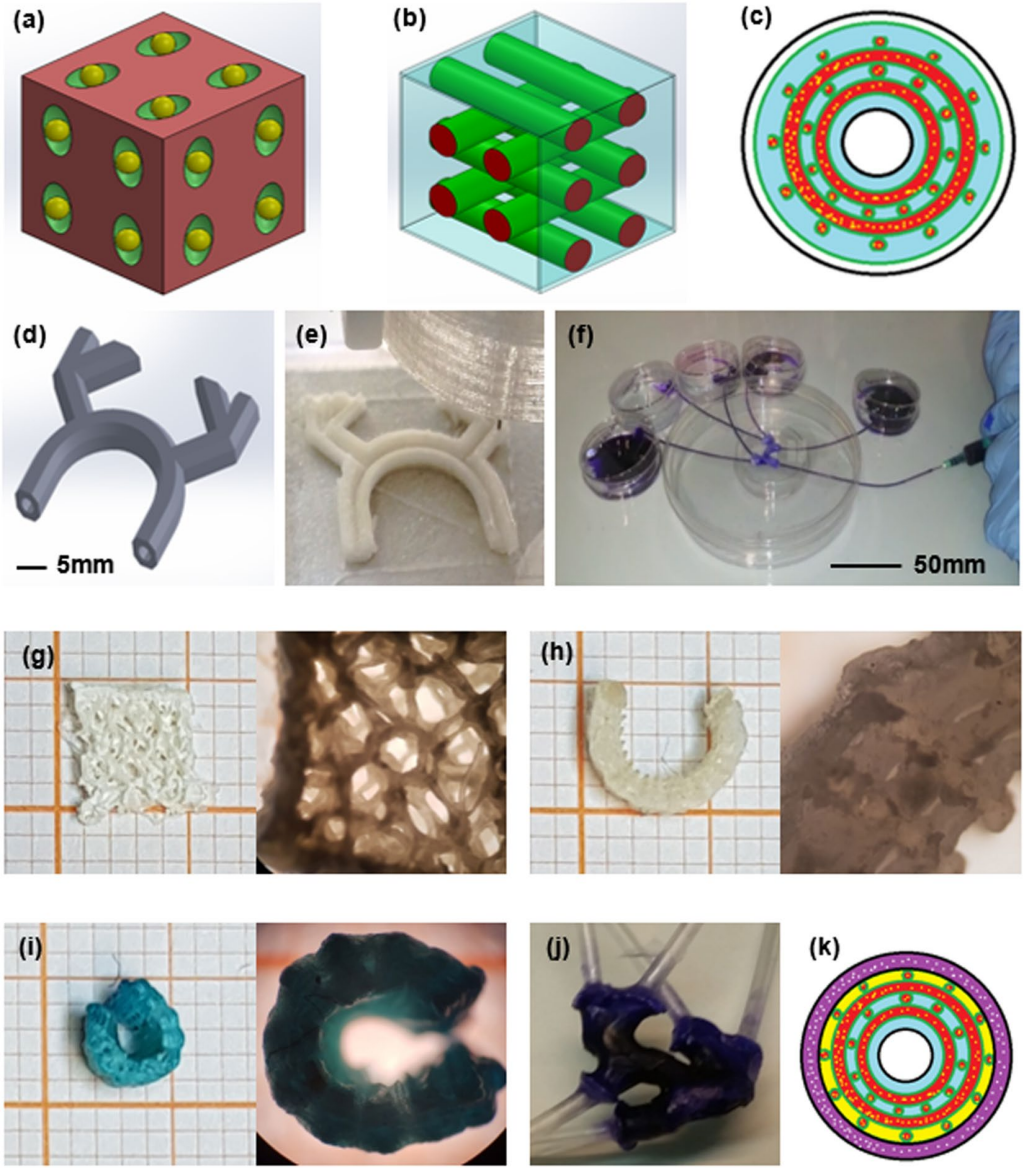
Bio-composite material vessel fabrication. (**a**) Composite biomaterial (bio-based, biocompatible and biodegradable): matrix (red, functions), interface (green, performance and stability) and reinforcement (yellow, fibre, nanoparticles, nano-tubes, etc.). (**b**) Bio-composite material (Nano-functionalised 4D bio-engineered scaffold): matrix (blue, functions), interface (green, performance and stability) and fibre (red, composite/biocomposite scaffold reinforcement).(**c**) Bio-composite material vessel schema: nano-laden fibre (yellow points and red), interface (green), not functionalised matrix (blue) and functionalised matrix (white). (**d**) 3D digital model of complex vessel. (**e**) Complex vessel section; (**f**) Nano-functionalised 4D Bio-engineered scaffold perfusion test. (**g**) Complex geometry aerogel scaffold (left panel) and related detail (right panel, 4x magnification). (**h**) Aerogel with functionalised surface micro-porosity (left panel) and related detail (right panel, 4x magnification). (**i**) Nano-laden bioengineered scaffold (left panel) and related detail (right panel, 4x magnification). (**j**) Dehydrated 5D bioprinted vascular device after more than 24 months of petri dish storage. (**k**) Bio-composite material customisation schema: nano-laden fibre (yellow points and red), interface (green), not functionalised matrix (blue), functionalised matrix for dedicated cells (yellow), adhesive coating (black) and customised matrix for physiology analyses (white point and violet).

The positive results obtained from *in-vitro* and *in-vivo* studies allowed us to move from functionalisation method (4D) to customisation method (5D). Then, we printed a model of a complex femoral artery bifurcation (Supplementary Fig. S3) directly from a patient’s computed tomography (CT)-scan with the aim to reconstruct, by 3D bioprinting, the portion of interest (Supplementary Fig. S4) with sodium alginate-based material. We functionalised the bio-ink with interface and reinforcement (composite biomaterial) (Fig. 6a), developing the required functionalisation for the proposed therapy or application. The obtained composite biomaterial was used to manufacture a new type of fibre, its interface and matrix, for bio-composite material (Fig. 6b) customisation with different functional matrix (cellular adhesion, gradual release, etc.) (Fig. 6c). Thus, merging this material with the correct technology parameterisation, we printed and perfused a complex vessel system (Fig. 6d–f).

The fibre (Fig. 6g) allows the scaffold engineering (performance mimicry, vascularisation for the cells viability); the surface micro-morphology modification (Fig. 6h) enables the different types of applications (cells adherence, active or passive stimuli response), and with different types of coating and matrix we can customise the 4D bio-composite materials^22^ (Fig. 6i). Moreover, to realise the nano-laden scaffold we can print a composite biomaterial or rehydrating an aerogel scaffold. After that, implementing the adhesive coating and the matrix we can customise the biological device (Fig. 6j) to achieve the desired physiological properties (Fig. 6k).

**Figure 7:**
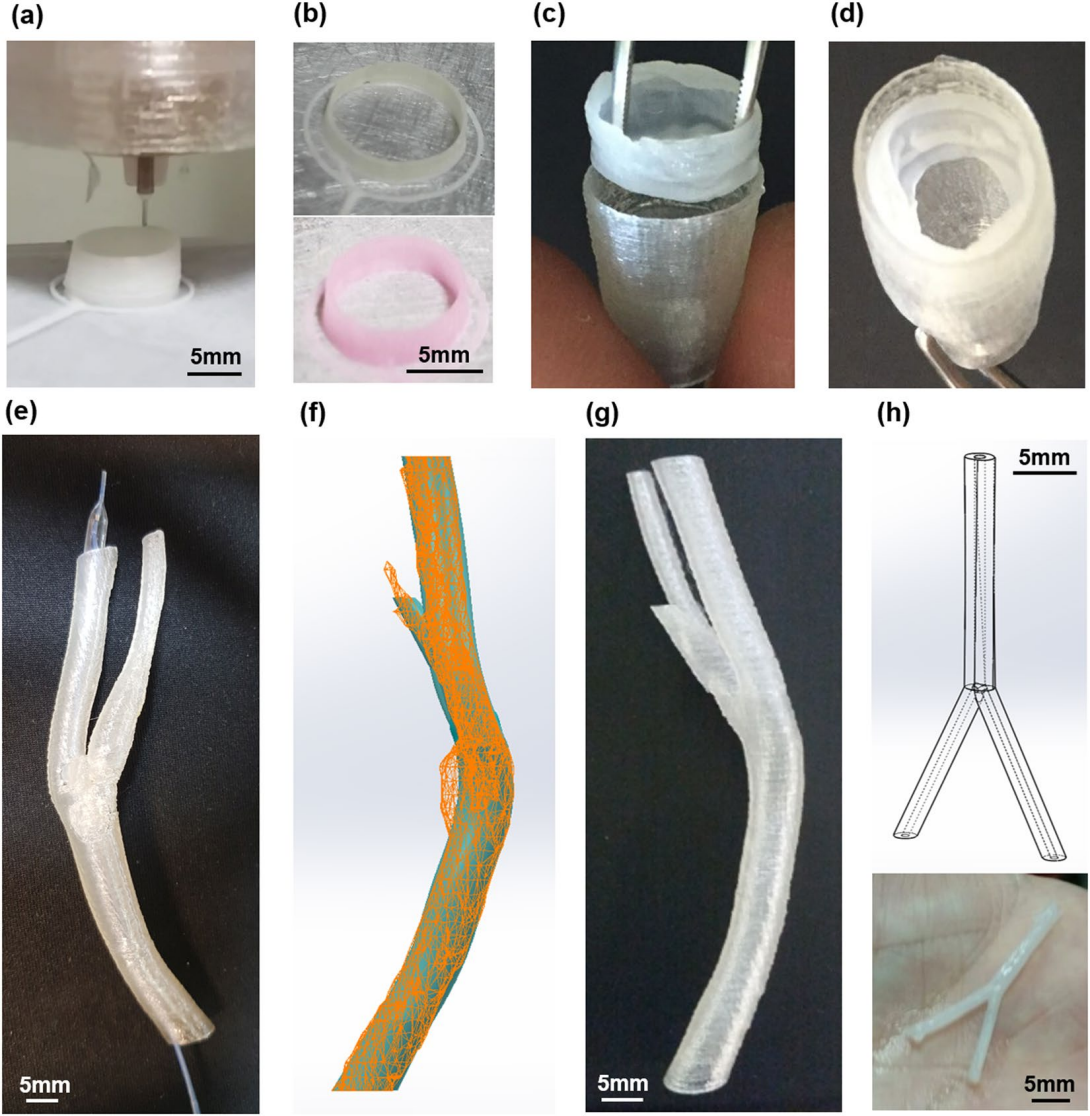
The 5D customised scaffold. (**a**) 5D soft scaffold printing phase. (**b**) Composite biomaterial scaffold with green (upper panel) and red (lower panel) fluorescent nanoparticle. (**c** and **d**) Integration of soft and hard materials for gradual release tests, respectively. (**e**) Femoral bifurcation model for STENT training. (**f**) Surfaces detail of not printable.STL model with 3D digital reconstructed model. (**g**) 3D PLA printed model without pathology. (**h**) Capillary vascular bifurcation with 0.65 mm inner diameter and 1.7 mm outer diameter, model design (upper panel) and 3D printed object (lower panel).

Finally, we obtained a 5D bioprinted device^12^ performing a customised coating balloon for the controlled release tests of new elements (Fig. 7a–d). The resulted fabrication process sets the basis for the post-printing phases and “6D” smart material device (i.e. autonomous regeneration), starting from the organ reconstruction for therapy customisation and the related training for the continuous improvement (Fig. 7e–g), to the controlled release systems for the functionalisation of long-term vascularised scaffold (capillary systems) (Fig. 7h).

## Discussion

The realisation of organs and living synthetic tissues requires biomimetic bio-papers (bio-scaffolds) for cell seeding, vascular systems for long-term perfusion^18^ and a precise cell deposition process with the related scaffold customisation for 5D technique implementation and biomimicry screening. For example, the macro-structure selection must permit the synthetic organ function biomimicry (i.e. vessel contraction) while the surface morphology modification should guarantee the cells adherence and the related viability.

Using the Formulation and Analysis for Nanoparticle Additive Manufacturing (FANNAM) method^15,23^ (Table 1) applied to the reconstruction of PAD, it is possible to carry out pre-surgical training^23,24^ and 5D short-term medical device, identifying the correlation and the related scheduling between methods steps, parameters, operative processes and the printing phases related to organ printing. The production of 5D printed soft tissues (cf. Table 1) requires: i) evaluation of the parameters that resume the interaction between the 3D printed object and the selected biological tissue, ii) functionalisation to apply operative processes in health application and iii) validation of the printing phases with 3D partial processes and customisation of digital bio-library. The 3D pre-printing process requires three major steps: image acquisition and curation, slicing and analysis, computer aided tissue engineering (CATE) processing and printing. Image acquisition is usually obtained with CT, due to its rapid sampling. Volumetric data are rendered in digital imaging and communication, in medicine format (DICOM), that cannot be 3D printed^25^. Thus, image data require conversion in stereolithography (STL) format, using a specialised software^26^, then the g-code generated by computer aided manufacturing (CAM) controls the motors. The selected material and its customisation address the selection of technology and the correct printing parameters for its manipulation. The process assuring the total quality improvement starts by identifying the type of object^27^ and the quality evaluation of the results for the adopted production technology and concerns: material properties, geometric parameters, micro-porosity, the degradation of the gelled object, as well as the vitality of the cells and/or the toxicity of the material. Rapid freeze prototyping technology enables the possibility to develop high defined scaffolds and the proposed method improves the current state of the art^28,29^. In fact, we reached the same resolution of existing technology^18,30^ but without the use of toxic photo-activators, indispensable for the stereolithographic apparatus (SLA). Moreover, compared to both selective laser sintering and SLA we do not have material waste and we can utilise cell-laden bio-ink, not possible with fused deposition modelling that reaches temperatures over 37 °C.

High resolution was achieved by identifying 46 geometrical parameters^18^ for the scaffold analysis, developing a continuous improvement operative process to better understand and validate the deviation trend and utilising the inner diameter value as “filament parameter”. Problems regarding the height of the layer were removed and variance using the geometrical parameters was reduced^28,31^ as well as the incomplete lap, the over accumulation and the redundant lap^32^. In this context, viscosity plays a pivotal role in the deposition of filament without support (bridge). In fact, it is well known that alginate dissolved in bovine serum affects the rheological properties of polymer mixture solution^33^ and the viscosity is directly related to the alginate concentration and is inversely related to temperature^34^. Indeed, the development of shape with high viscosity material becomes very easy in printing, however the energy that has to be subtracted from the scaffold and/or the time for the freezing phase has to be added.

In order to identify the region of interest, defined by the material, we generated a digital model and the related digital bio-library able to control the manufacturing phases. This library requires a lot of validation since we can apply the same file for its development with the higher number of hard and soft materials.

For the reasons exposed above it is mandatory that the identification of macro-morphology resolution is merged with the printing parameters lower limits. The FANNAM method includes four processes: 3D reconstruction cutting (3DRC), 3D fast freeze gelation (3DFFG), nano dry formulation (NDF) and 3D partial processes (3DPP), designing new digital elaboration steps to define the best correlation between different materials and technology. The final aim is the definition of an excellent printing configuration^17^ and an alginate-based material formulation with embedded NPs, simultaneously disinfecting the material. With 3DRC we are able to reconstruct the missing biological components, defining the best fit between 3D model and final trajectory related to printing materials. Alginate based materials with NPs, used for these experiments, were made with NDF, without NPs waste and aiming to ensure low variance in the evaluation of released nano-laden material. We implemented 3DFFG that permits the fast gelation, the release of alginate in a very short time (approx. 2–3 minutes) and simultaneous disinfection^35^, enabling the long-term preservation without any added treatment. In fact, once the adherent printed object is obtained with the digital model, the instantaneous gelation closed the loop for scaffold fabrication, fixing the freezed scaffold instantaneously.

The functional adherence of the object, the amount of material used and similarity with the digital model are assured via the 3DPP that includes all activities of configuration, calibration or modification carried out in the processes and stages of 3D model development, which competes for maximum quality performance. Partial processes are therefore mandatory in the development of objects through rapid prototyping and viscous materials. For example, changing the development speed on a machine with a subtractive technology, the surface roughness alteration does not modify the mechanical characteristics, but using the additive manufacturing technology to realise cake decorations, its morphology changes, with the functional aim of the object (aesthetics). Thus, the assessment of the parameters is directly related with the functionality of the 5D object and in the case of the soft tissue development we have to take in consideration drops accidentally falling down or the creation of vascular channel to bring, where required, the gelation fluid for the morphology long-term fixation and dedicated tests^33,34,36–41^ are required to better explore any manufactured object and each possible customisation.

Finally, post-print and analyses increase the quality and the biological potentials of digitalised pathological models and the evaluation of the printed 5D model adherence assuring a continuous improvement system with the use of a custom CATE for the smart and fast storing of all processed data in new digital library pathology.

Thanks to the high scaffold customisation potential (NP or functionalised matrix for a gradual drug release), we can design and bioprint 5D personalised medical devices. In detail, modifying the composite biomaterial^42–45^ and the manufacturing methods, the interaction^45,46^ of the transplanted structures with the biological substrate can be varied accordingly to experimental needs. When it is possible to graft biocompatible objects (prosthesis, STENT, etc.) and activate the functionalised material for restoring physiological activity with, for example, external stimuli (electrical, magnetic, photonic, etc.) or chemical agents, the 5D printing becomes “active”^14,47,48^. Thus, thanks to the analyses and the digitalisation of the diseases over the time, it is possible to validate and determine key directives in order to develop the region of interest for the scaffolds for future *in-vivo tests* and Food and Drug Administration/European Medicine Agency (FDA/EMA) draft about nano-functionalised 5D printed devices.

This method can be applied to different types of material and gelation, and we utilised alginate, gelling the scaffold with EtOH and CaCl_2_. The two developed biomaterial scaffolds resulted in very different micro-morphology as well as functional application but both showed low impact on cell death. Dissolving the nano-laden scaffold in a solution with VSMC and subsequently into the rat vena cava, the developed devices demonstrated the ability to control gradual release, very similarly to STENT applications, the vehiculation of NPs and long-term storage potential. Reproducing the same tests on HUVECs, the results demonstrated the potential of human scaling of the proposed method. The high variability in biological application requires rigorous standards for analysis and we identified a good scheduling for the 5D processes and parameters digitalisation with the aim to support the synthetic organ development and the digital bio-library improvement. Our method showed that is possible to develop nano-laden 5D devices for training, pharmacological tests and *in-vivo* applications, improving the typical drug eluting balloon therapy merging patient morphology with the high resolution of rapid freeze prototyping technology.

## Materials and Methods

### 3D bioprinter, thermal analysis and model design

The rapid freeze prototyping bioprinter (Bio-4esti)^18^, placed under a biosafety cabinet (HeraSAFE Heraeus, Thermo Fisher scientific, Waltham, MA, USA) is equipped with two mechanical extruders^24^ for 5 ml syringes (BD Emerald, Franklin Lakes, NJ, USA) and a removable Peltier cold plate. The cold plate temperature was measured with a Flir A325 infrared camera (Flir Systems, Wilsonville, OR, USA). The analysis of the thermal behaviour was carried out using the application FLIR ResearchIR v.1.2 (Flir Systems, Wilsonville, OR, USA). With this software tool, it is possible to evaluate the minimum, the maximum and the average temperatures of a selected rectangle of an image, or a movie, taken by the camera. In our case, the rectangle was fitted on the cold plate.The scaffold was designed using SolidWorks2015 (Solidsolution, London, UK) and sliced with Slic3r open source software.

### Nano-functional scaffold development

In our experiment two different materials are used: alginate powder^18^ (W201502, Sigma-Aldrich, Steinheim am Albuch, Germany) that is a low-cost hydrosoluble biomaterial used for sweets fabrication or for molecular cuisine, and natural polylactic acid (PLA)^11^ (175N1, Velleman Inc., Legen Heirweg, Gavere, Belgium) without colour pigments (diameter 1.75 mm (1/16′), density 1.25 g/cm^3^ (at 21.5 °C), printing temperature 190–225 °C, impact strength 5 kJ/m^2^) which recently demonstrated its use for clinical applications^49^.

#### NDF

Immediately before the experiments, in order to minimise particle aggregation, all NPs (40 nm Fluorescent nanobeads, Thermofisher, Milano, Italy) were sonicated (Branson Ultrasonics, Danbury, CT, USA) for 30 mins at T = 37 °C and 3 µl (50 µg/ml) of NPs solution were added into sodium alginate powder. Subsequently, high glucose DMEM w/o Phenol Red (Gibco, Thermo Fisher Scientific, Waltham, MA, USA) was added to achieve a final alginate solution concentration of 7%, 9% and 11% wt/vol.

#### 3DFFG

Scaffolds were printed onto aluminium plate with a temperature of about −30 °C (at room temperature), using a 26 G needle (BD Emerald, Franklin Lakes, NJ, USA) and covered by ethanol 95%, for the simultaneous gelation and disinfection^50,51^ phases. In detail, the nano-laden scaffolds for the *in vitro* and *in vivo* analyses were developed with alginate 11% at 6 mm/s of printing speed.

### Micro-porosity characterisation

In order to obtain morphological information on the 3D printing scaffolds, SEM characterisations were performed using a field emission SEM (FESEM, Nova NanoSEM 450, FEI company, Hillsboro, OR, USA). Images were acquired in field-free lens mode using the Everhart-Thornley detector for the secondary electron imaging signal. The accelerating voltage of 10 kV, the spot size of 4.5 nm and the working distance of 6 mm were used in the acquisition of all images.

### *In-vitro* VSMCs analysis and dissolution test

#### Cell culture

Mice VSMCs were kindly provided by Prof. Leonardo Elia (Istituto Clinico Humanitas Rozzano, Milano, Italy). VSMC was cultured in DMEM (high glucose) with 2 mM glutamine, 10% fetal bovine serum (FBS, Euroclone, Milano, Italy) and 1% penicillin/streptomycin (5,000 U/mL). Cells were cultured as recommended and maintained under standard cell culture conditions at 37 °C in a water-saturated atmosphere of 5% CO_2_ in air. We seeded 3×10^5^ VSMCs in a 50 mm petri dish for direct and indirect measurement (n = 8). The direct measurement consists in adding after 24 hrs to the culture the alginate scaffold-enriched NPs (50 µg/ml) crosslinked with ethanol, as described previously. In regards to the indirect method, the same scaffold-enriched NPs were totally dissolved directly in the medium before being added to the culture.

#### Viability assay

To evaluate cell viability, VSMCs were counted in a Bürker hemocytometer by trypan blue exclusion method and evaluated under a phase contrast microscope. The viability of cells was also confirmed using calcein AM staining (Thermo Fisher, Waltham, MA, USA). Specifically, after the treatment the media was replaced with a fresh one containing 5 µM Calcein AM. After 30 mins of incubation at 37 °C in 5% of CO_2_, cells were washed twice with phosphate buffered saline (PBS) before being imaged with an upright fluorescent microscope (Leica Microsystem, Wetzlar, Germany) through a x20/0.7 or ×40/1.3 oil objective.

#### Scaffold dissolving methods

We performed two different dissolving methods: direct and indirect.

1. Direct method: the scaffold was placed directly in the petri dish containing cells with 4 ml of culture medium.
2. Indirect method: the scaffold was placed and dissolved in a 15 ml tube into 2 ml of culture medium; once the scaffold was dissolved, were added 2 ml more of culture medium and then this solution was added in the petri dish, where the cells were previously cultured.

### Experimental animals

Two Sprague Dawley Rats have been used in these experiments accordingly to the local ethical guideline and the protocol was approved by the Italian Ministry of Health (Prot. N 989/2017-PR). All the procedures followed the directives of the European Law 63/2010 and the Italian law 26/2014 for the experimental animal use. Briefly, we anesthetised animals as previously described^52^ and the vena cava was exposed after surgery. The upper part of the vena cava was ligated and the NPs-laden scaffold was inserted after a 1 cm distal to occlusion small incision. We left the scaffold for 2 minutes in the vena and then we rapidly removed this area, washed in PBS, longitudinally opened and fixed in 4% buffered formalin solution for 24–48 h till two-photon microscopy evaluation.

### *In-vitro* and *In-vivo* microscope analysis

#### STED microscopy

The cells were fixed on a cover glass (thickness n°1.5) with 4% paraformaldehyde (PFA) for10 mins, for the STED microscopy, blocked, and permeabilised with 3% normal goat serum, 0.1% Triton X-100 in 1 x PBS for 1 hour as previously described^53^. Briefly, STED xyz images were acquired with a Leica SP8 STED3X confocal microscope system (Leica Microsystem, Wetzlar, Germany). Green NPs (Molecular Probes, Eugene, OR, USA) were excited with a 488 nm Argon Laser and emission was collected from 507 to 614 nm, while wheat germ agglutinin (WGA, Thermo Fisher, Waltham, MA, USA) was excited with a 545/547nm-tuned white light laser (WLL) and emission was collected from 555 to 647 nm. Sequential acquisition was applied to avoid fluorescence overlap. A 660 nm conventional wisdom (CW)-depletion laser was used for both excitations. Images were acquired with a Leica HC PL APO 100×/1.40 oil STED White objective. CW-STED and gated-STED were applied to the fluorescent NPs and WGA, respectively. Collected images were de-convolved with Huygens Professional software (Scientific Volume Imaging, Hilversum, Netherlands) and analysed using Imaris 7.4.2 software (Bitplane, Belfast, UK).

#### Two-photon microscopy

Rat vena cava subjected to NPs-laden scaffolds has been fixed and stained with WGA, as previously described^53^, and imaged with the two-photon microscopy (Trim Scope II, LaVision BioTec, Bielefeld, Germany) which allow to imaging cell and NPs for the entire thickness with a 10–20 µm of Z-stack.

### *In-vitro* HUVEC analysis

The sterile plastic material for the cell cultures was purchased from Costar, Corning (Amsterdam, The Netherlands), and PBS from Euroclone (Milano, Italy). The ATP colorimetric assay kit was obtained from Novus Biologicals (Centennial, CO, USA). MTT was provided by Sigma (St. Louis, MO, USA), which also supplied all of the other reagents unless otherwise specified.

#### Cell culture, treatment and proliferation/viability studies

HUVECs (purchased from Lonza, Basel, Switzerland) were grown in a fully supplemented EGM-2MV Bullet Kit (Lonza, Basel, Switzerland) at 37 °C in a 5% CO_2_ humidified incubator, and used at passages 3–5 in the experiments. Before the treatments, cells were seeded in plates and cultured to 80–90% confluence.

Following, we seeded 3×10^5^ of HUVECs in 2 ml of medium for 2–24–48 hrs before toxicology assays, applying the direct/indirect treatment previously used for the VSMCs.

The morphology of the cells was monitored using an inverted microscope (Olympus CK40-RFL, Tokyo, Japan) and their number was evaluated by cell counting in a Bürker hemocytometer.

Viability was also assessed by (3-(4,5-dimethyl-thiazol-2-yl)2,5-diphenyl tetrazolium bromide) MTT assay. The formazan crystals were solubilised, and the absorbance was measured using an automated microwell plate reader (Multiskan Ascent, Thermo Labsystems, Helsinki, Finland) at 550 nm.

Cellular ATP levels were determined using the ATP assay kit, following the manufacturer’s instructions.

All results were expressed as the percentage of controls (untreated cells).

The uptake of fluorescent NPs was evaluated by flow cytometry using a FC500 flow cytometer (Instrumentation Laboratory, Bedford, MA, USA). Data were processed using the FlowJo software package (Tree Star Inc., Ashland, OR, USA).

### Statistics

Normal distribution of variables was checked by means of the Kolmogorov-Smirnov test. Statistics of variables included One sample T-test signed rank test, unpaired Student’s t-test, two-way ANOVA (post hoc analyses: Bonferroni test or Games-Howell test, when appropriate) Wilcoxon sign rank test and Kruskal-Wallis (post hoc analyses: Dunn’s multiple comparison). Prism 6.0 software (GraphPad Software, San Diego, CA, USA) was used to assess the normality of the data and for statistical calculation. The details on the specific test used for each experiment are reported in the figure legends. Statistical significance was set at p < 0.05.

## Supplementary information

Supplementary Figures
