# Author Correction: *In-vivo* vascular application via ultra-fast bioprinting for future 5D personalised nanomedicine

**DOI:** 10.1038/s41598-020-69893-0

**Published:** 2020-10-19

**Authors:** Ruben Foresti, Stefano Rossi, Silvana Pinelli, Rossella Alinovi, Corrado Sciancalepore, Nicola Delmonte, Stefano Selleri, Cristina Caffarra, Edoardo Raposio, Guido Macaluso, Claudio Macaluso, Antonio Freyrie, Michele Miragoli, Paolo Perini

**Affiliations:** 1 Department of Medicine and Surgery, University of Parma, via Gramsci 14, 43126 Parma, IT Italy; 2 CERT, Centre of Excellence for Toxicology Research, via Gramsci 14, 43126 Parma, IT Italy; 3 Department of Engineering and Architecture, University of Parma, Parco Area delle Scienze, 43124 Parma, IT Italy; 4 Unit of Surgical Sciences, Azienda Ospedaliero-Universitaria, via Gramsci 14, 43126 Parma, IT Italy; 5 Centro Universitario di Odontoiatria, University of Parma, Via Gramsci 14, 43126 Parma, IT Italy; 6 IMEM-CNR National Research Council, Parco Area delle Scienze 37/A, 43124 Parma, IT Italy; 7 Unit of Vascular Surgery, Azienda Ospedaliero-Universitaria, via Gramsci 14, 43126 Parma, IT Italy; 8 Humanitas Clinical and Research Center – IRCCS, via Manzoni 56, 20089 Rozzano Milan, IT Italy

Correction to: *Scientific Reports* 10.1038/s41598-020-60196-y, published online 21 February 2020

The original version of this Article contained an error in Affiliation 8, which was incorrectly given as ‘Humanitas Clinical and Research Centre, via Manzoni 56, 20090, Rozzano Milan, IT, Italy.’. The correct affiliation is listed below:

Humanitas Clinical and Research Center – IRCCS, via Manzoni 56, 20089, Rozzano Milan, IT, Italy

This error has now been corrected in the HTML and PDF versions of the Article, and in the accompanying Supplementary Information file.

