# Supplementary material for: In-vivo vascular application via ultra-fast bioprinting for future 5D personalised nanomedicine
Source: Sci Rep. 2020 Feb 21;10:3205. doi: 10.1038/s41598-020-60196-y (PMC7035336; doi:10.1038/s41598-020-60196-y)
Supplement: Supplementary file 1 — Supplementary Figures [file 41598_2020_60196_MOESM1_ESM.pdf]

In-vivo vascular application via ultra-fast bioprinting for future 5D personalised nanomedicine

Ruben Foresti*[,1,2], Stefano Rossi[1,2], Silvana Pinelli[1], Rossella Alinovi[1], Corrado Sciancalepore[3], Nicola Delmonte[3], Stefano Selleri[3], Cristina Caffarra[1], Edoardo Raposio[1,4], Guido Macaluso[1,5,6], Claudio Macaluso[1], Antonio Freyrie[1,7], Michele Miragoli[1,2,8] and Paolo Perini[1,7]

1) Department of Medicine and Surgery, University of Parma, via Gramsci 14, 43126 Parma (IT)
2) CERT, Centre of Excellence for Toxicology Research, via Gramsci 14, 43126 Parma (IT)
3) Department of Engineering and Architecture, University of Parma, Parco Area delle Scienze, 43124 Parma (IT)
4) Unit of Surgical Sciences, Azienda Ospedaliero-Universitaria, via Gramsci 14, 43126 Parma (IT)
5) Centro Universitario di Odontoiatria, University of Parma, Via Gramsci 14, 43126 Parma (IT)
6) IMEM-CNR National Research Council, Parco Area delle Scienze 37/A, 43124 Parma (IT)
7) Unit of Vascular Surgery, Azienda Ospedaliero-Universitaria, via Gramsci 14, 43126 Parma (IT)
8) Humanitas Clinical and Research Centre, via Manzoni 56, 20090 Rozzano Milan (IT)

*Corresponding author: Ruben Foresti, ruben.foresti@unipr.it

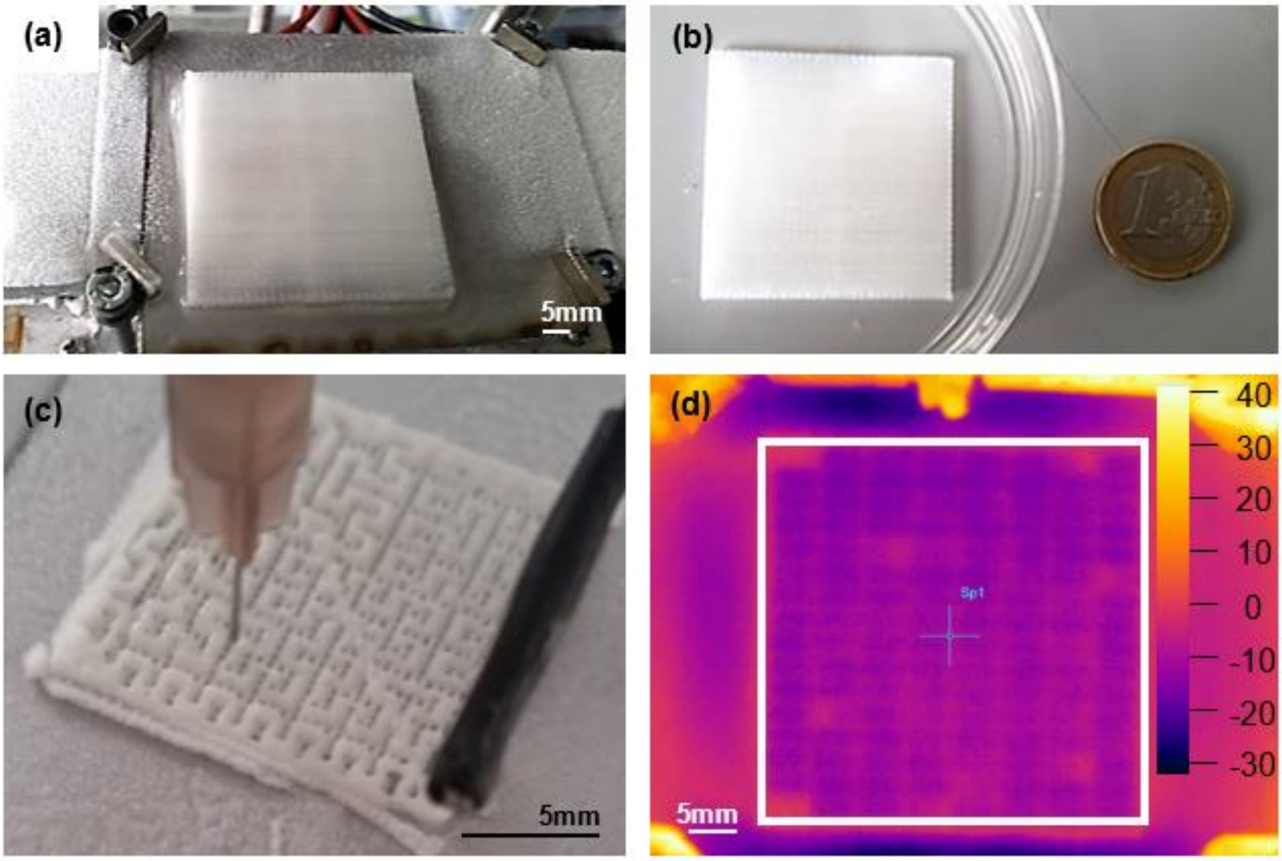

Supplementary Fig. S1

Peltier cold plate and bioscaffold fabrication: (a) Bioscaffold with 200µm of macro-porosity on the Peltier cold plate. (b) Detail of 47.5x47.5mm scaffold. (c) Hybrid scaffold with Hilbert curve geometry. (d) Thermal camera visualisation of the Peltier cell and detail of electro-thermal transition, the white square identifies the analysed surface area.

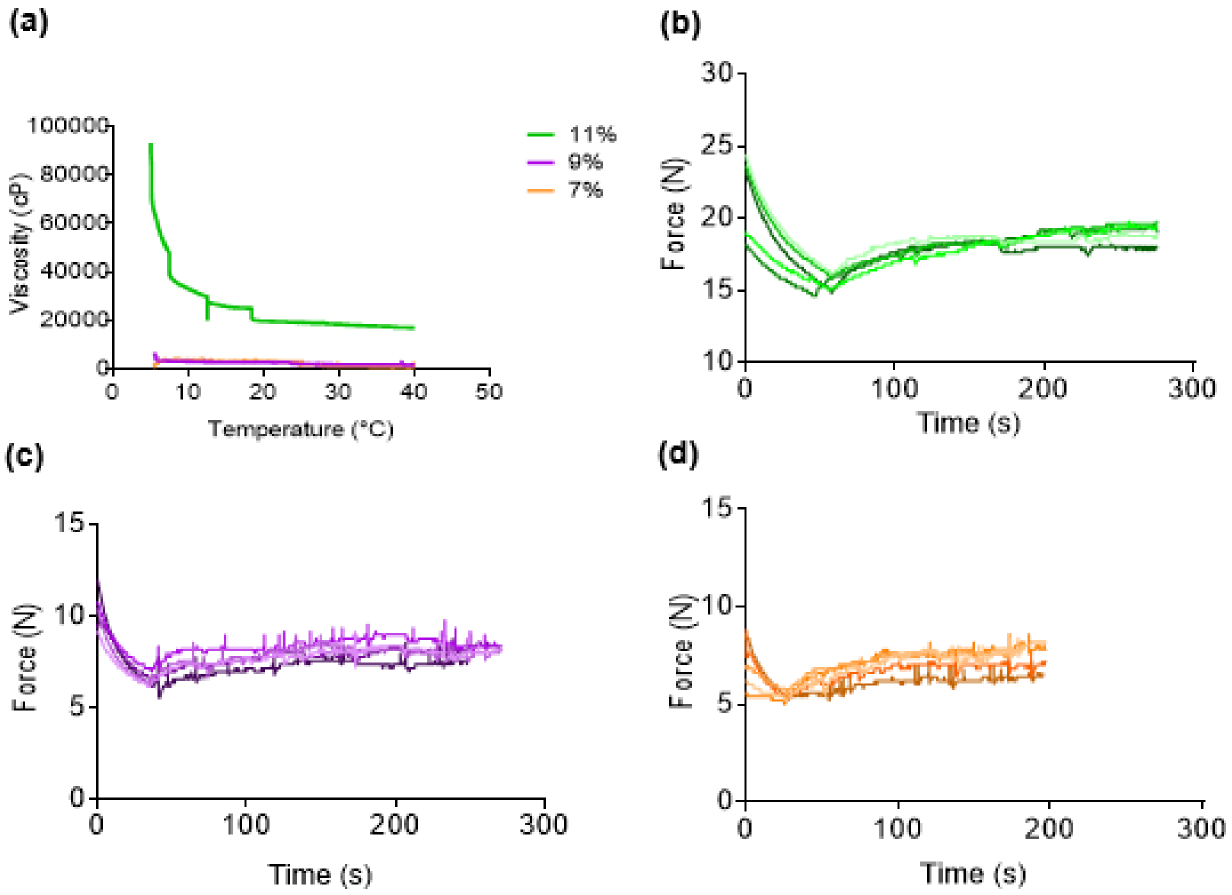

Supplementary Fig. S2

Viscosity and extrusion force applied to the syringe for three different alginate percentage scaffold fabrication. (a) Temperature vs viscosity relationship of different bio-ink alginate percentage (green 11%, purple 9% and orange 7%). (b) Force applied to the syringe for printing 5 different scaffolds with a speed of 20 mm/s and 11% of alginate concentration. (c) same as (b) with 9% of alginate concentration. (d) same as (b) with 7% of alginate concentration.

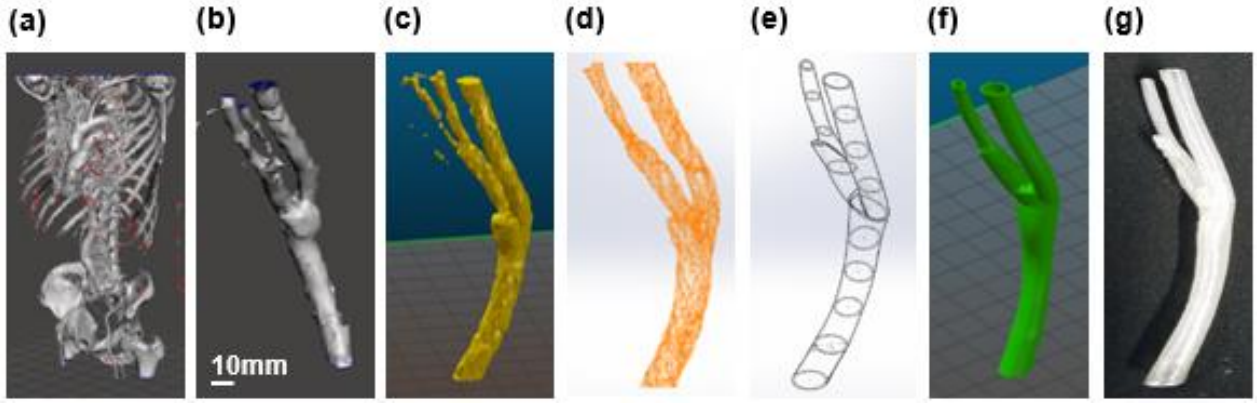

Supplementary Fig. S3

The 3D Reconstruction Cutting of a femoral artery bifurcation. (a) CT model file. (b) Section of femoral bifurcation. (c) .STL format. (d) Surfaces detail of not printable .STL model. (e) 3D digital reconstructed model. (f) .STL format of printable model. (g) 3D printed PLA femoral bifurcation.

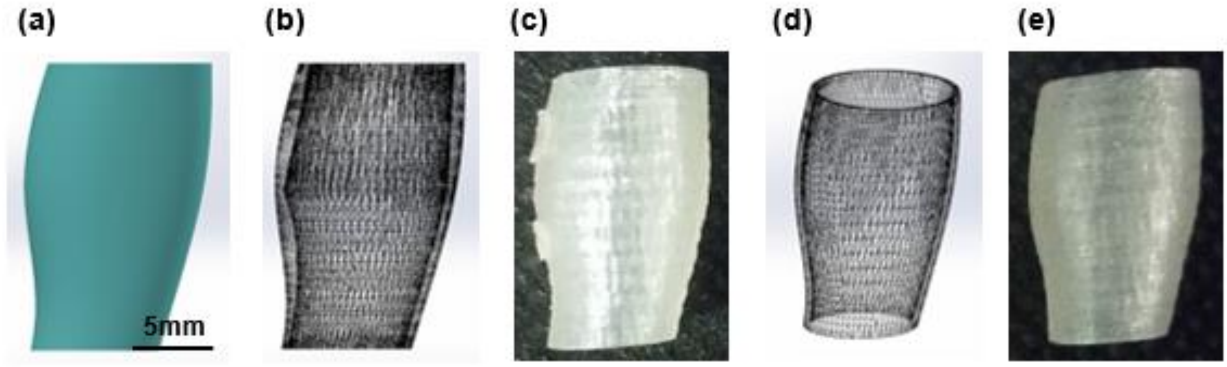

Supplementary Fig. S4

The 3D Partial Processes. (a) A part of a 3D femoral section model. (b) Automatic .STL generated by the CAM. (c) 3D printed PLA object with defects. (d) Adapted .STL model. (e) 3D printed object without defects and scalable for soft tissue bioprinting.
